# P-961. Let’s Shake on It: Evaluation of Weekly Handshake Antimicrobial Stewardship Rounds with Hospital Internal Medicine Providers

**DOI:** 10.1093/ofid/ofaf695.1163

**Published:** 2026-01-11

**Authors:** Drew T Dickinson, Dan Ilges, Sandhya Nagarakanti, Jamilah Shubeilat, Maria Teresa A Seville

**Affiliations:** Mayo Clinic Arizona, Phoenix, Arizona; Mayo Clinic Arizona, Phoenix, Arizona; Mayo Clinic Arizona, Phoenix, Arizona; Mayo Clinic Arizona, Phoenix, Arizona; Mayo Clinic Arizona, Phoenix, Arizona

## Abstract

**Background:**

Handshake antimicrobial stewardship (HAS) is an effective stewardship approach characterized by regular, face-to-face interactions with antimicrobial prescribers and broad review of all active antimicrobials. Benefits of this approach include enhanced relationship building and opportunities for targeted education. We conducted a mixed methods study to evaluate the impact of once-weekly HAS rounds among Hospital Internal Medicine (HIM) teams in an academic medical center.

**Methods:**

An infectious diseases (ID) physician and pharmacist conducted weekly table rounds with a HIM teaching team consisting of a hospitalist, advanced practice providers (APPs), APP fellow(s), and sometimes medical students. All patients on antimicrobials and without an active ID consult were reviewed and recommendations were provided. Thereafter, the stewardship team solicited *ad hoc* questions from other HIM teams via walking rounds in their offices. The number, type, and acceptance rate of recommendations were collected prospectively. A survey was sent to all HIM providers to obtain feedback on HAS rounds.

**Results:**

HAS rounds were conducted weekly from February 7, 2024, to February 19, 2025, with a total of 52 sessions. Over the study period, 340 recommendations were provided, and 314 recommendations were implemented, yielding a recommendation implementation rate of 92.4%. The most common recommendation types were antimicrobial discontinuation (n=76, 22.4%), duration modification (n=63, 18.5%), and de-escalation (n=39, 11.5%). The survey was sent to 102 HIM providers and completed by 19 for a response rate of 18.6%. All respondents stated HAS added value or greatly added value to their practice. Antimicrobial selection (n=16, 84.2%), de-escalation (n=12, 63.2%), and pharmacy expertise (n=6, 31.6%) were areas in which providers saw the greatest benefit. Eighteen providers (94.7%) recommended expansion of HAS to non-HIM services.

**Conclusion:**

Targeted, once-weekly HAS rounds resulted in high-yield interventions that were frequently accepted and perceived as highly valuable by HIM providers. Once-weekly or intermittent HAS practices might be considered in practice settings where resources or logistical constraints limit broader, hospital-wide handshake stewardship practices.

**Disclosures:**

All Authors: No reported disclosures

